# Single-Cell Dynamics Reveals Sustained Growth during Diauxic Shifts

**DOI:** 10.1371/journal.pone.0061686

**Published:** 2013-04-30

**Authors:** Sarah Boulineau, Filipe Tostevin, Daniel J. Kiviet, Pieter Rein ten Wolde, Philippe Nghe, Sander J. Tans

**Affiliations:** FOM Institute AMOLF, Amsterdam, The Netherlands; Baylor College of Medicine, United States of America

## Abstract

Stochasticity in gene regulation has been characterized extensively, but how it affects cellular growth and fitness is less clear. We study the growth of *E. coli* cells as they shift from glucose to lactose metabolism, which is characterized by an obligatory growth arrest in bulk experiments that is termed the lag phase. Here, we follow the growth dynamics of individual cells at minute-resolution using a single-cell assay in a microfluidic device during this shift, while also monitoring *lac* expression. Mirroring the bulk results, the majority of cells displays a growth arrest upon glucose exhaustion, and resume when triggered by stochastic *lac* expression events. However, a significant fraction of cells maintains a high rate of elongation and displays no detectable growth lag during the shift. This ability to suppress the growth lag should provide important selective advantages when nutrients are scarce. Trajectories of individual cells display a highly non-linear relation between *lac* expression and growth, with only a fraction of fully induced levels being sufficient for achieving near maximal growth. A stochastic molecular model together with measured dependencies between nutrient concentration, *lac* expression level, and growth accurately reproduces the observed switching distributions. The results show that a growth arrest is not obligatory in the classic diauxic shift, and underscore that regulatory stochasticity ought to be considered in terms of its impact on growth and survival.

## Introduction

In the presence of two carbon sources, bacterial cells may either metabolize them both at the same time, or first use one and then the other. The latter strategy has been termed diauxic growth [1]. A classical example is the growth of *E. coli* on a mixture of glucose and lactose, which is characterized by initial rapid growth on glucose, followed by a phase of arrested growth when glucose is depleted, until the *lac* enzymes are expressed that allow growth on lactose [2]. Studies of glucose-lactose diauxie have led to many key discoveries on biological regulation, ranging from the existence of regulatory proteins and operator regions [3] to catabolite repression [4]. More generally, nutritional shifts experiments have revealed the dynamic changes of key classes of cellular components, such as protein, DNA, and ribosomes [5], [6], [7], [8], [9]. However, our understanding of the cellular growth response to environmental change has been obtained primarily using bulk techniques [7], [9], [10], [11], [12], [13] that measure the growth rate of the population as a whole. As a result, it is unclear how the growth of individual cells responds during diauxic shifts.

This question is central to understanding how cells compete. Cellular heterogeneity within populations could critically affect the ability to consume limited resources before they are exhausted by competitors, which can be decisive for survival. For instance, populations could respond fast by following a bet-hedging strategy, in which the expression of genes is randomly turned on, thus generating sub-populations that are primed for diverse future environmental changes [14]. On the other hand, stochasticity in regulatory control could be disadvantageous, as the costs of spuriously expressing genes may lower the rate of growth and reproduction [15]. Stochasticity in gene regulation may thus have important consequences for fitness, and therefore, shed a new light on the function of regulatory systems in complex natural environments, as well as their historical evolutionary origins.

The advent of single-cell techniques has in recent years quantitatively characterized the stochastic nature of gene expression [16], [17], [18], [19]. The *lac* system in particular has been shown to display stochasticity in expression [19], as well as in the underlying repressor-operator association and dissociation events [20]. In response to changes in artificial inducer, *lac* expression was shown to exhibit bistability [21], [22] and heterogeneity in the timing of induction [23]. However, it remains poorly understood how cellular growth is affected. To address this issue, we have studied the dynamics of diauxic growth at the single-cell level. We used a microfluidic approach to control glucose and lactose levels in the cellular environment, accurately determined cellular lengths at high time resolution, and used GFP labeling to monitor expression of the *lac* operon.

## Results

### Population Growth and Expression Dynamics during Diauxie

**Figure 1:**
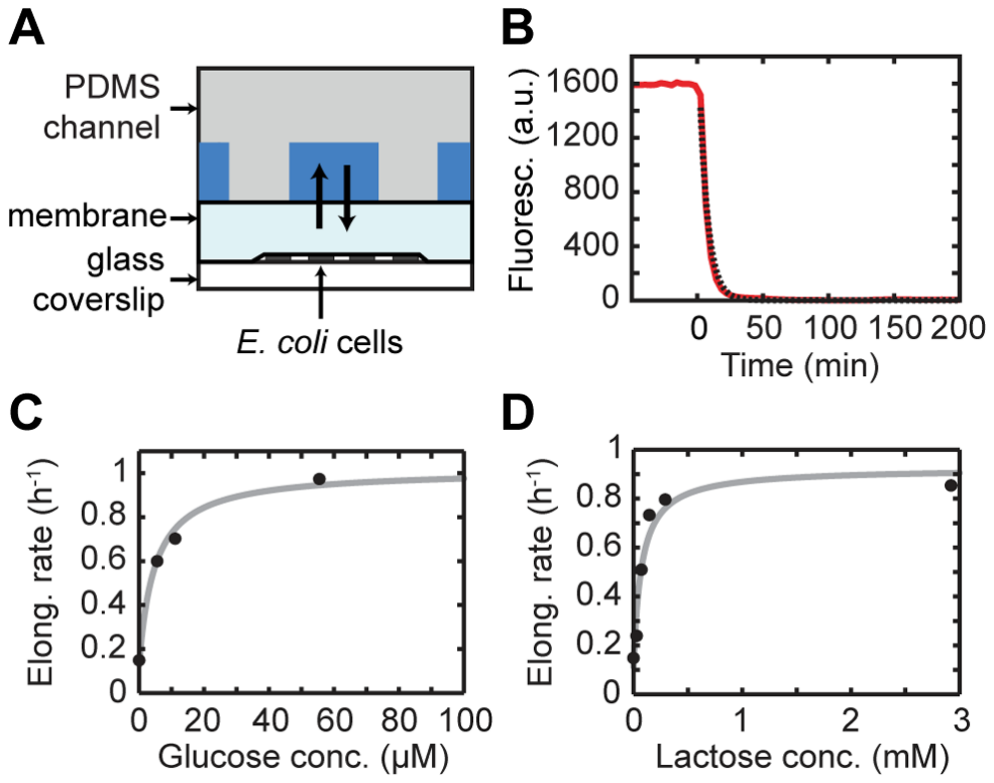
Experimental setup. (A) Layout of the microfluidic device. The cells are growing between a glass coverslip and a polyacrylamide gel membrane. The medium reaches the cells and is exchanged by diffusion through the membrane. (B) Estimation of the fluid exchange time by means of the fluorescent glucose analog 2-NBDG (30 µM). 2-NBDG is removed at time zero. The experimental curve is shown in red; the exponential decay fit is shown in black. (C–D) Mean growth rate of *E. coli* in steady-state growth in minimal media containing glucose (C) or lactose (D) as the only carbon source. In both cases, the fitted line is a Monod growth curve taking into account a non-zero growth rate on contaminants.

To control the environment, we used a microfluidic device in which microcolonies of cells were growing between a membrane and a coverslip [24], [25] (Figure 1A and Material and methods). By flowing different media above the permeable membrane, cells were exposed to a variable but spatially uniform environment. We measured the depletion of a fluorescent glucose analog from the cellular area, upon instantaneous switch to plain minimal medium, and found an exponential decay with a half-life of ∼5 min (Figure 1B). Starting with one or two cells, growing microcolonies were monitored by phase-contrast microscopy for 8–9 generations, yielding 200 to 500 cells for each microcolony at the end of the experiment. The lengths of the cells were determined using phase contrast images acquired every 1 to 2 min, and custom image analysis software (Material and methods and Text S1). We started by characterizing the steady-state growth limitations, using fixed external nutrient concentrations (Figure 1C and D and Figure S1 and S2). The data for both glucose and lactose were consistent with the Monod relation [1], indicating characteristic limiting concentrations of 5 µM for glucose and 70 µM for lactose. For glucose a value of ∼1 µM has been found previously in batch cultures [26]. Induction with artificial inducers IPTG and TMG has been shown to lead to bistability in *lac* expression [20], [21], [22]. Here we did not observe bistability, thus confirming theoretical predictions that natural inducers do not give rise to bistability because they are actively degraded by metabolism [27], [28].

**Figure 2:**
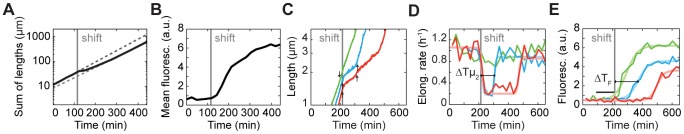
Dynamics at the population level and in single cells. (A) Growth curve for a typical microcolony, indicating the sum of all cell lengths within the colony. (B) Mean fluorescence intensity (per unit area) within cells, averaged over a microcolony. (C) Single-cell length over time for three different lineages, representing cases with no growth rate decrease (green), a lag phase (blue) and a longer lag phase (red). Arrows indicate cell division events. The curves are vertically shifted for clarity. (D) Elongation rates obtained by exponential fits to the length data at sub-cell cycle resolution. Drawn lines are fitted parameterized functions. ΔTµ_2_ is the time difference between the time of shift and the half maximum to growth recovery after shift. (E) Fluorescence levels for the three lineages in (C) and (D). Drawn lines are fitted parameterized functions. ΔT_F_ is the time difference between the time of shift and the half maximum to induction after shift. Black bar: 120 min before the shift, over which data was averaged to determine the expression level prior to the shift.

We then subjected cells to an environmental switch after 4–5 generations. Switching from glucose to lactose or from a mixture of glucose and lactose to lactose only, gave similar results as expected (Figure S3). We used a starting glucose concentration at which the growth rate is maximal (555 µM, see Figure 1C). The population growth, quantified by adding the lengths of all cells within the microcolony, displayed the prototypical diauxic growth behavior (Figure 2A): first a phase of rapid exponential growth, followed by a lag phase, after which growth restores to exponential growth. The two exponential growth rates matched the values for the fixed glucose and lactose media (∼1 doubling/hr, and ∼0.8 doubling/hr respectively). The duration of the lag phase was about 20 min, comparable to batch culture data [2], [29]. Expression of the *lac* operon was monitored using a GFP reporter (see Material and methods). The mean fluorescence intensity within the microcolony in the first exponential phase was near the cellular autofluorescence, consistent with expected repression of the *lac* operon when growing on glucose [2]. Upon the shift, the mean fluorescence increased and reached a steady state level on a timescale that is similar to that observed for the growth rate (about 300 min, Figure 2B). The fluorescence started to increase rapidly 30 min after the shift, though the precise onset of expression increase could not be determined precisely because the rise was smooth. Overall, these observed population dynamics of growth and expression are consistent with previous results from batch experiments.

### Growth Dynamics in Single Cells during Diauxie

We followed the growth of individual cells during the diauxic shift by determining their length over time. The instantaneous growth rate was determined at sub-cell cycle resolution by fitting the cell length over time to an exponential function (see Text S1). We observed no significant changes in cell width during the experiment. Cells within one colony displayed diverse growth behaviors (Figure 2C and D). For the majority, traces of length versus time showed a sharp transition from the glucose growth rate (1.0 doubling/hr) to a low growth rate (between 0.2 and 0.6 doubling/hr), followed by a smoother transition to the lactose growth rate (0.8 doubling/hr). The low growth rate was similar to growth in media without any added carbon sources, which we could observe either in constant conditions (Figure 1C and D), or when switching from glucose to a medium without added carbon sources (Figure S4). These experiments suggested the intermittent growth at low rate was supported predominantly by metabolism of contaminants in the media, which unlike in batch cultures are continuously replenished in these experiments. Other minor contributions to growth after the shift could potentially come from internal cellular glucose reserves, residual glucose that was not depleted, and from lactose metabolized by leaked *lac* enzymes produced at low repressed levels. The length analysis sometimes displayed measurement artifacts at cell division, but their amplitude was small compared to the general trend and therefore did not affect the growth analysis (Figure 2C, red). The data thus showed that the lag phase did manifest itself at the single-cell level.

**Figure 3:**
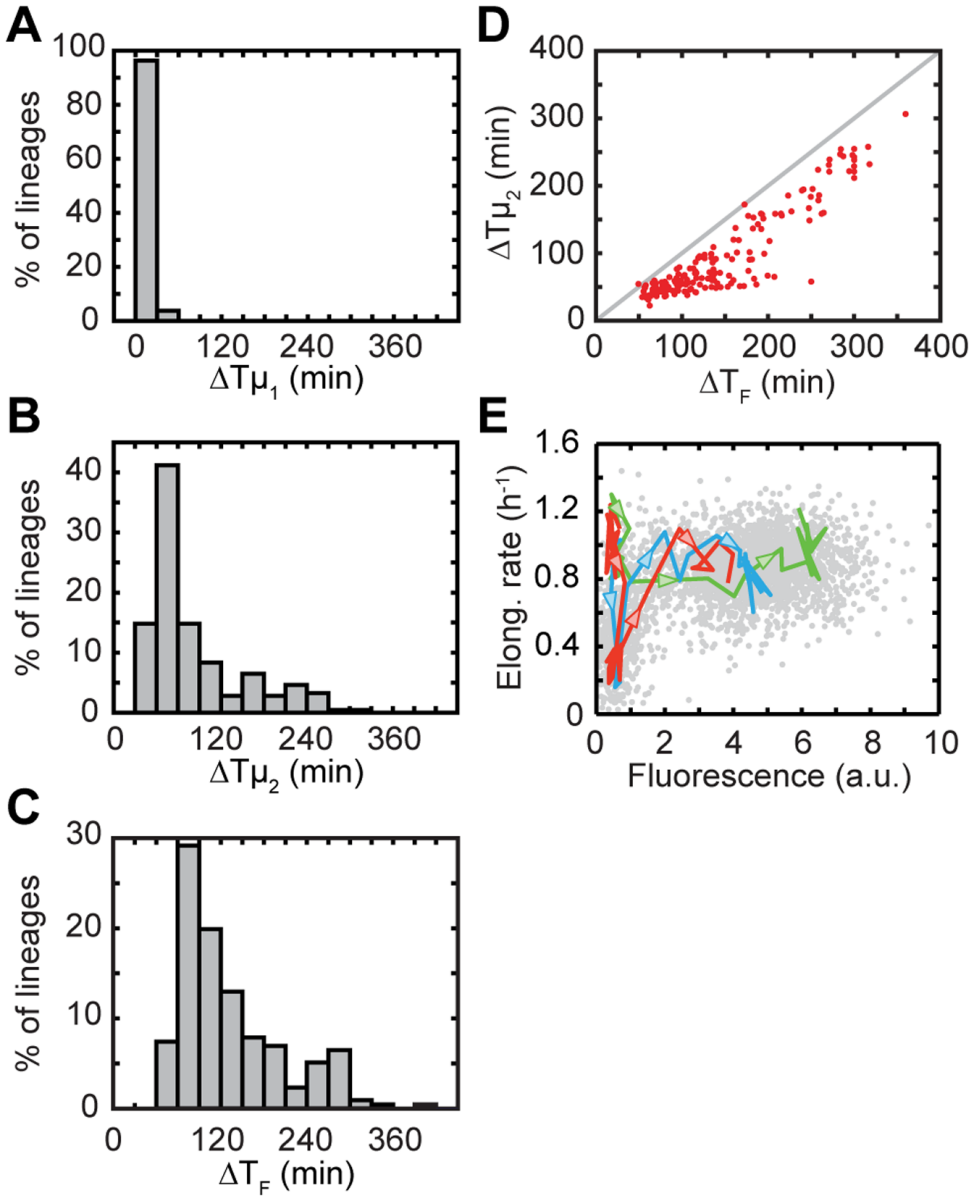
Statistical analysis and correlation between expression and growth. (A) Distribution of growth decrease times ΔTµ_1_. (B) Distribution of growth recovery times ΔTµ_2_. (C) Distribution of fluorescence recovery times ΔT_F_. N = 216 for all histograms. (D) Scatter plot of the delays in growth recovery versus delays in fluorescence increase for 185 cell lineages. r^2^ = 0.85, p-value <0.001. The line drawn is ΔTµ_2_ = ΔT_F_. (E) Elongation rate versus internal *lac* levels for all lineages (scatter plot, in grey) and the three lineages in Figure 2C. Arrows are directed towards increasing time.

To characterize the variability in growth dynamics, we determined the moment of growth decrease (ΔTµ_1_) and restoration (ΔTµ_2_). Both are quantified relative to the moment of switching the fluid flow, which we refer to as the shift time. The difference between ΔTµ_2_ and ΔTµ_1_ is a measure for the duration of the lag phase in individual cells. We found that ΔTµ_1_ and the duration of the lag phase are not significantly correlated (Figure S5), suggesting that the growth decrease and the growth restoration are independent processes. ΔTµ_1_ was narrowly distributed close to zero (mean of the distribution: 13 min), which shows the growth process responds rapidly to the glucose decrease (Figure 3A). ΔTµ_2_ on the other hand displayed a broad and asymmetric distribution that extended to lag times of up to hundreds of minutes (79 min on average) (Figure 3B). A small fraction of lineage, ∼5%, even failed to resume exponential growth within the timescale of the experiment. These delays thus exceed by far the average lag time of 20 min. This broad distribution of lag times suggests that escape from lag is strongly affected by the timing of stochastic internal cellular events. We did not find correlations between progression into the cell cycle and the timing of the growth transitions (r^2^≈0.03, p-value = 0.02 for growth decrease; r^2^≈0.001, p-value = 0.7 for growth recovery, N = 185).

Interestingly, not all cells showed a lag phase. Some cells displayed no discernible decrease in growth rate (15% of all traces) (Figure 2D), in contrast with the abrupt entry into lag seen in the other cells. The absence of a lag phase did not appear to be related to correlations with the cell cycle, or the position of the cell within the microcolony (Figure S6). The ΔTµ_2_ distribution extended monotonically down to zero, which suggested that the cells without lag do not represent a distinct sub-population (Figure 3B). To understand the origins of this lack of a lag phase in some cells, information on the dynamics of *lac* operon expression is required.

### Correlations between Growth and Expression in Single Cells

We determined the mean fluorescence per unit area within single cells as a measure for the *lac* operon expression. The fluorescence versus time for individual lineages had a sigmoidal shape: a low level close to the background during growth on glucose, followed by a rise some time after the shift to lactose, until a constant steady-state level was achieved on the order of the doubling time (Figure 2E). However, different lineages displayed significant variability. For instance, the fluorescence level at the end of the experiment varied by up to 40% (see Figure 2E), reflecting heterogeneity in protein production between cells [19] as well as incomplete entry into steady state for some lineages. This final fluorescence level did not correlate significantly with the timing of induction (r^2^ = 0.04 and p-value = 0.003, N = 216).

The timing of induction was also variable, as observed previously for the *ara* system [30]. The time between the shift and the moment at which fluorescence reaches half-maximum, which we here denote as ΔT_F_, was distributed with a width and shape similar to that of ΔTµ_2_ (133 min on average) (Figure 3C). We found ΔT_F_ and ΔTµ_2_ to be strongly correlated (Figure 3D, r^2^ = 0.85, p-value <0.001). This correlation is directly evident in the individual traces (Figure 2C–E), where the red lineage displayed both a long growth arrest and a long induction delay, while the blue lineage with a smaller growth arrest exhibited a correspondingly smaller induction delay. The correlations also indicated that ΔT_F_ was systematically larger than ΔTµ_2_, which we will address below. Overall, these data are consistent with a simple model in which the lag phase is caused by the *lac* operon being in the repressed state, and exit out of the lag phase is triggered by the stochastic *lac* induction.

However, this model did not explain the sustained growth. In particular, at the moment of glucose exhaustion (ΔTµ_1_) the *lac* operon in these cells was still ‘off’, with expression at low repressed levels (N = 31, mean fluorescence 15 min after the time of shift: 0.76±0.34 (SD) a.u.). While we did not observe spatial heterogeneity of nutrients, for instance when using the fluorescent glucose, we cannot formally exclude that some spatial differences in the precise moment of glucose exhaustion occur. However, delayed glucose exhaustion should merely delay all events, including the moment at which the repressible effect of glucose is alleviated (catabolite repression). Hence, while spatial nutrient inhomogeneity could lead to growth arrests occurring at different times for different cells, it does not explain the absence of a growth arrest. The fluorescence traces also showed no sign of *lac* bistability, where the *lac* operon spontaneously switches between repressed and induced expression levels, as has been observed for artificial inducers that are not metabolized [21], [22], [23]; bistability is thus excluded as the cause of sustained growth. It has also been shown that the moment of *lac* induction upon a change in the artificial inducer TMG depends on the *lac* expression level before the change [23]. Hence we wondered whether the leaky stochastic expression of the repressed *lac* operon [19], [31], could underlie the variability in growth responses. If so, the *lac* expression level before the shift should correlate with ΔT_F_. However, such a correlation is difficult to detect, as the measured expression level during glucose growth is similar to the autofluorescence of wild-type cells. Nonetheless, the mean fluorescence 120 min before the shift did exhibit a weak but significant correlation with ΔT_F_ (r^2^≈0.08 and p-value <0.001, N = 216). This result suggests that the sub-population of cells exhibiting sustained diauxic growth originated from pre-existing variations in expression that had developed stochastically during glucose growth.

While the data supported the idea that expression variability caused the observed differences in the growth dynamics of individual cells, a number of questions remained unanswered. For instance, how can the low leaky expression when glucose is exhausted be sufficient to maintain the growth rate at high levels, and why does growth restoration seem to precede induction? The latter is seen by ΔT_F_ being systematically larger than ΔTµ_2_ by up to hundreds of minutes (Figure 2D), and hence cannot be explained by the ∼10 min GFP maturation time [30]. To address these questions we have developed a stochastic model, which is detailed in the next section.

### Stochastic Model of Diauxic Growth

**Figure 4:**
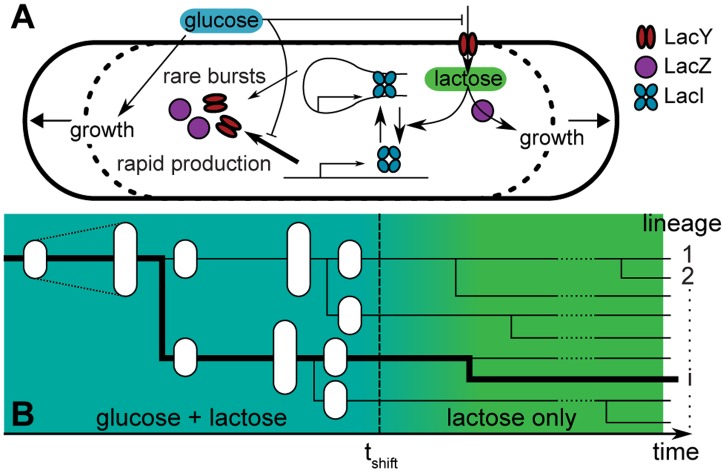
Stochastic model. (A) Within each cell the concentrations of lactose, LacYZ and LacI are simulated, as well as the operator state. Lactose imported from the environment or glucose lead to cell growth. (B) Each cell is simulated until it reaches a specified length, at which point it divides to produce two daughter cells. The proteins of the parent cell are partitioned randomly between the two daughters. The daughters are then simulated until their subsequent division. Growth and fluorescence recovery times (T_F_ and T_µ_) are extracted from the reconstructed cell lineages.

**Figure 5:**
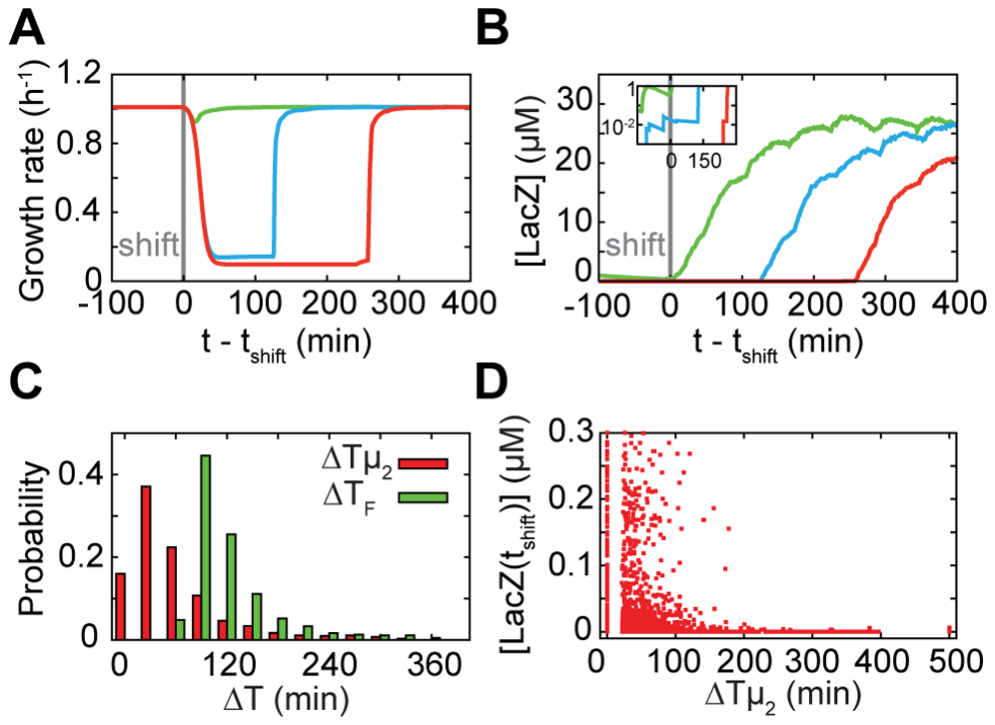
Results of the stochastic model. (A) Example time-series of cell growth rate for a cell with fast (green), slow (blue) and very slow (red) response. (B) Fluorescence time-series for the cells shown in (A). Inset: The same data on a logarithmic scale, showing that cells with higher expression levels at the time of shift of medium tend to be induced more rapidly. (C) Histograms of growth (red) and fluorescence (green) recovery times, ΔTμ_2_ and ΔT_F_. In panels (C) and (D), cells at ΔTμ_2_ = 0 showed a decrease in growth rate of less than 20%. (D) *Lac* expression of each lineage at t_shift_ plotted against growth recovery time. Cells which did not reach the induction threshold in the time of the simulations are placed at ΔTμ_2_ = 500 min. Cells with initial concentrations above ∼10 nM typically have a rapid recovery of growth rate. Note that the plot range does not represent the full range of initial expression levels.

Diauxic growth has been studied extensively using mathematical models [32], [33], [34], [35], [36]. With the aim to gain intuitive insight into the main features of observed heterogeneity, we developed a minimal stochastic model that focuses on key features and neglects various known details (Figure 4A; see Text S1 for complete description). For instance, for simplicity we considered the stochastic binding and dissociation of repressor at a single operator site and neglected other operator sites, as well as stochasticity arising from variabilities in the *lac* repressor levels [23]. The free and bound operator states yielded respectively a high and low rate of stochastic transcription events, with the latter resulting from brief partial repressor dissociations. Cellular metabolism and growth depended on *lac* expression through lactose import and metabolism, following deterministic Michaelis-Menten kinetics and using the experimentally determined dependence of growth on glucose (Figure 1C). In turn, *lac* expression depended on metabolism through the deactivation of free LacI repressors and random dissociation of DNA-bound repressors stimulated by intracellular lactose. Inducer exclusion and regulation by the cAMP pathway were modeled phenomenologically. Cells divided at a specified size, and their contents were randomly partitioned between the two daughters. Parameter values for the various reactions were, where possible, taken from direct experimental measurements; otherwise, these were inferred indirectly or fit to available experimental data (see Text S1 for details). An initial population of 100 cells was simulated for 210 min on both glucose and lactose, after which external glucose decreased exponentially (decay time τ = 5 min, Figure 4B). Delay times ΔTµ_1_, ΔTµ_2_ and ΔT_F_ were determined using the same criteria as for the experimental data. Overall, we found growth and *lac* expression dynamics to be similar to the experiments (Figure 5A–C). While most cells showed sharply decreased growth around 20 min after the shift, a small fraction of the cells did not and instead maintained a high growth rate (∼15% of cells, green trace, Figure 5A). Note that the simulated growth rates do not account for random perturbations of the growth rate from other sources as observed in the experiments (∼20% of the mean growth rate), and are thus artificially smooth. The moment of growth restoration was again highly variable, with ΔTµ_2_ and ΔT_F_ distributed similarly as for the experiments (Figure 5C).

Analysis of the temporal dynamics revealed how growth rates could be maintained at high levels during diauxie. First, stochastic leaky expression of the *lac* proteins during glucose growth gave a fraction of cells a somewhat higher *lac* protein concentration just before the shift, while still at repressed levels [20], [31] (see Figure 5B and D). The resulting comparatively high lactose import triggered the start of induction of the *lac* operon. However, when external glucose becomes exhausted in these cells, the *lac* expression and hence the concentration of LacZ enzymes that can metabolize lactose is still near repressed levels. A non-linear dependence of growth on *lac* expression is therefore essential as a third ingredient, while metabolism of contaminants and residual glucose may also provide small contributions to the overall growth rate. The relation between cellular growth and expression can here be studied directly by plotting the fluorescence intensity against the concurrent instantaneous growth rate (Figure 3E). The data after growth restoration shows that induction to just 20% of fully induced levels is sufficient to induce near maximal growth on lactose. Thus, even low *lac* levels can generate high growth rates, which is essential to sustaining growth at high levels during the transition. The steep dependence also explains the observed delays of induction with respect to growth for the cells with a lag phase (Figure 3D): growth reaches a near-maximal rate as enzyme production is only beginning to be ramped up.

The inherent positive feedback in the system is crucial for the rapid escape from the lag phase: LacY permeases allow lactose to enter the cell, which in turn leads to *lac* induction and hence increased numbers of permeases. We find that the threshold level of transporters required to initiate this positive feedback is small, with just a few *lac* transporters at the time of shift sufficient for a rapid induction within ∼90 min (see Figure 5D and Figure S7). On the other hand, cells without permease at the time of shift exhibit a very broad distribution of induction delays (mean ΔT_F_≈180 min), with a significant probability of not being induced at all within the time-frame of the simulations. The major component of this delay is waiting for a first stochastic burst of production caused by a partial repressor dissociation (mean waiting time of ∼200 min), consistent with a previous study of *lac* induction kinetics [20] and a recent theoretical model [37]. Dissociation of repressor typically follows rapidly (within about 20 min), such that expression can be induced to the threshold level (in about 50 min) (see for example Figure 5A and B, red trace, around 250 min). We note that the high threshold for the number of *lac* transporters required for induction reported in a recent study [20] was related to the bistability of that system and the intermediate amounts of inducer that were added, which can explain the difference with our observations. We find that two mechanisms counter this escape from lag: metabolism of lactose and dilution of lactose by volume expansion both decrease its concentration, which tends to drive cells back towards the off expression state, and ultimately arrest of growth. However, both metabolism and dilution are comparatively slow during the lag phase, and hence even a small number of permeases can maintain an appreciable internal concentration of lactose. Once full induction is achieved the import rate is sufficiently fast to support a high internal lactose concentration, making repressor rebinding rare, and the induced rapid growth state stable.

### Genealogical Relations

**Figure 6:**
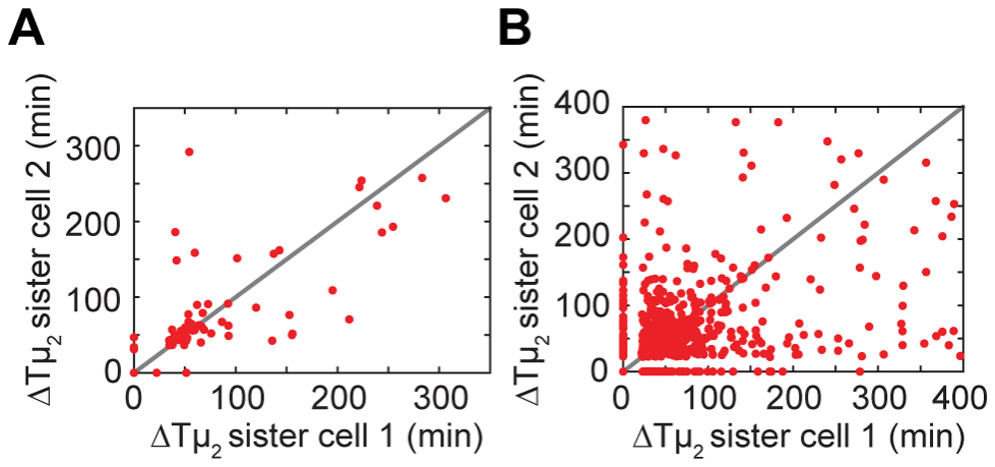
Switching synchrony of sister cells. The growth recovery delays ΔTµ_2_ are plotted for pairs of sister cells. (A) Data obtained from experiments. N = 75, r^2^ = 0.52, p-value <0.001. (B) Data resulting from simulations. N = 660, r^2^≈0.13 and p<0.001. Note that in both cases lineages in which one cell switches but its sister or its progeny does not are not plotted (in total: 22 pairs for the experimental data, 146 pairs for the numerical data).

Our results indicated that expression history can determine the timing of future switching events. This non-genetic cellular ‘memory’ could result in correlated behavior between genealogically related cells [38]. To test this possibility we compared the delay in growth recovery (ΔTµ_2_) of a recovering cell with the recovery delay of its sister (or, if this sister does not recover, the sister’s progeny). We find a weak but significant correlation (r^2^ = 0.52, p-value <0.001, Figure 6A). A control with randomly picked pairs of recovering cells does not display any correlation (r^2^ = 0.008, p-value = 0.47). The simulations also show a weak correlation between growth recovery times (Figure 6B). One may wonder what causes this correlation between sister cells, as the gene expression bursts that underlie exit from the lag phase are stochastic, which should make them independent and uncorrelated. However, a newborn cell inherits *lac* enzymes expressed by the mother, intracellular lactose, as well as *lac* repressors, which all affect the escape probability from the *lac*-repressed state. In particular, both daughter cells will inherit a similar propensity for repressor dissociation and hence full induction. The persistence of correlations between sisters for lag times up to 250 min is surprisingly long, but consistent with the expected decorrelation time for protein concentrations, which are much longer in the lag phase due to the slow growth and volume expansion (the doubling time during lag phase can be up to 5 hours). Additionally, the time between the division event generating the two sisters and induction, which is the time available for decorrelation of the two sister lineages, can be much shorter than the overall lag time if the sister cells divided after the shift of medium. If a cell divides after a small production burst but before dissociation of the repressor, which typically takes tens of minutes after such a production burst, then both daughter cells are likely to inherit some of the *lac* proteins and a significant level of lactose. The two daughter cells will therefore both be induced shortly after division, resulting in very similar values of ΔTµ_2_ for the two daughter lineages.

## Discussion

Monod’s original glucose-lactose diauxic growth assays have become the prototypical illustration of the regulation of gene expression. Not only did they reveal the underlying molecular mechanisms, but also how growth and survival in complex environments – the essential cause of their evolutionary origins – is impacted. In the last decade, novel methods to monitor single cells over time has highlighted the stochastic nature of gene expression and its causal molecular mechanisms, and has allowed us to begin uncovering its impact on signal propagation and differentiation [39], [40]. But how molecular stochasticity of biological systems affects their growth and survival remains poorly understood [41], [42]. Here we aimed to begin addressing this problem by interrogating how stochasticity in *lac* expression impacts the dynamics of growth, upon switch from glucose to lactose on single-cell level.

We found that although a population as a whole may display diauxic lag, a significant fraction of the cells within this population (∼15%) does not and thus produces an immediate growth response. This result counters the common notion that the speed of the response to lactose changes is determined by the processes of *lac* operon induction, protein dilution and degradation [43]. While correct for the transcriptional response, this study shows that growth responses can be much faster. Our experiments showed cells maintaining a continuously high growth rate, uninterrupted by the switch from glucose to lactose detection and metabolism. Paradoxically, the expression of the *lac* enzymes importing and metabolizing lactose was repressed at the time of shift, and turning on their expression required of order 100 min – conditions that seems more consistent with an obligatory lag phase. However, stochastic simulations together with experimental correlations between expression and growth pointed to a plausible explanation: the stochastic basal *lac* expression before the shift provided some cells with limited but sufficient *lac* permeases to achieve rapid induction, which in turn yielded sufficient *lac* catabolic enzymes to support near-maximal growth rates on lactose by the time glucose was depleted. Limited permease levels were sufficient owing to the positive feedback between *lac* expression and the lactose import rate. Furthermore, the steep dependence of growth on *lac* enzyme concentration permits rapid growth shortly after induction and well before full expression levels are reached. We note that while this model accurately predicts the central experimental features, it is a minimal one, and additional mechanisms can be considered. For instance, one could imagine that growth during the switch is supported rather by other compounds such as acetate that are produced by metabolism overflow during growth on glucose [36]. However, this seems unlikely as such compounds will be depleted at the same rate as glucose by the external flow. Growth could also be briefly supported by internally-buffered compounds [44], though this should not result in the observed heterogeneous growth dynamics.

While a minor fraction of cells continues to grow after the sugar switch, another fraction of the population displayed surprisingly long lag phases. Lag times often exceeded the doubling time as well as the population average lag time by several fold. We showed that these long delays are consistent with the experimentally-observed timescale of rare bursts of *lac* expression in the repressed state, combined with an expression threshold that must be crossed for the ‘on’ state to remain stable [20]. The stringent response has been shown to be involved in diauxic shifts [45], [46]. Thus stochasticity in cellular components associated with the stringent response, such as ribosomes, could be another source of variability in the growth response, in addition to variability originating from *lac* operon expression. Our results further put a different perspective on the gradual exit from stationary phase as observed at the population level. Exit from the lag phase is significantly more abrupt for individual cells, with the gradual exit seen in bulk assays stemming from averaging over cells with a wide distribution of lag times.

The findings have implications for competition and survival when resources are limited. In general, cells may follow various strategies in heterogeneous environments [41], [47]. One option is responsive switching, in which cells detect the changes in the environment and thus can change their phenotype appropriately. The advantage is that all cells within a population can deterministically exploit new opportunities, but at the cost of expressing a sensing machinery [15], and of time delays involved in changing a cellular phenotype. While the cellular response may be affected by noise in the sensing machinery during and after the environmental change, any cellular heterogeneity prior to the change does not play a role. In contrast, in the stochastic switching strategy [41], cells in constant conditions continually switch in a stochastic manner between phenotypes, and the population as a whole thus displays many different phenotypes. Here, cells are not burdened by sensing costs, yet some cells will be well-adapted directly and thus can act upon opportunities without delay. However, there are significant costs in spuriously expressing phenotypes that are not utilized, and not all cells within the population can exploit transient opportunities.

The data presented here suggest a hybrid third option, namely a stochastic sensing strategy, which overcomes the central tradeoffs. Here, a stochastic expression of the sensing machinery allows a fraction of the population to respond deterministically, without delay, at minimal costs. While the *lac* repressor - a sensor in the *lac* system - is constitutively expressed in line with a responsive strategy, the *lac* transporter also plays a central sensing role, and it is expressed stochastically at low repressed levels in the absence of lactose. As a result, some cells respond immediately while others respond slowly as they wait until the expression of their sensing machinery randomly gets turned on. The costs for the rapidly responding cells are limited, as the sensing function of the *lac* enzymes - nutrient detection - requires just a fraction of transporters that are expressed at full induction. Note that while it is weak, a tradeoff does remain, as the slowly responding cells express even less of the sensing machinery in glucose. Importantly, even the metabolic function of the *lac* enzymes - nutrient import and catalysis - initially requires just a fraction of full induction because of the non-linear expression-growth relation. This enables immediate growth responses with zero delay, despite the delays involved in turning expression up until fully induced levels. Rapid growth responses are particularly acute when competing for limited resources: those genotypes capable of responding rapidly may consume all resources before slow genotypes respond, and hence dramatically out-compete them. Stochasticity is an essential ingredient in this strategy, as it limits the burden of maintaining the responsive state to just a fraction of the population, and thus hedging its bets at minimal cost on future episodes when lactose becomes available.

## Materials and Methods

### Strain and Media

All experiments were performed with the *E. coli* strain AB460 (created by A. Böhm and kindly provided by M. Ackermann). AB460 is a derivative of MG1655 (*rph-1 ilvG- rfb-50*). To measure the expression of the *lac* operon, *lacA* was replaced with *GFPmut2* [48] and chloramphenicol resistance using the protocol described by Datsenko and Wanner [49].

Cells were grown in M9 minimal medium (47.7 mM Na_2_HPO_4_, 25 mM KH_2_PO_4_, 9.3 mM NaCl, 17.1 mM NH_4_Cl, 2.0 mM MgSO_4_, 0.1 mM CaCl_2_) (all the chemicals were provided by Merck), with 0.2 mM uracil (Sigma), supplemented with 0.01% (w/v) glucose (Merck) or 0.1% (w/v) lactose (Fluka). The M9 medium supplemented with lactose also contained cells with a knocked-out *lac* operon (NCM520, obtained from the Coli Genetic Stock Center). These cells cannot grow on lactose but can grow on the contaminants present in the medium. NCM520 cells were inoculated from glycerol stock in M9+0.1% (w/v) glucose for growth overnight. The following day, cells were washed with M9+0.1% (w/v) lactose and transferred to the same medium to be used for experiments. To control for glucose depletion, the red fluorescent dye sulforhodamine 101 (0.01 mg/mL) was systematically added to the M9 medium containing glucose.

### Growth Protocol

Cells were initially inoculated from glycerol stock in TY medium and grown until the OD >0.02 and next diluted in M9 medium with 0.01% (w/v) glucose for growth overnight. The following day, the overnight culture was diluted in M9 medium with 0.01% (w/v) glucose (OD ∼0.005) and transferred to the microfluidic chamber. 10 µL of culture were deposited on a glass coverslip, the polyacrylamide gel membrane was put on top and left to dry for about two minutes before the setup was assembled. All these steps were performed at 37°C.

### Microfluidic Device Fabrication

The microfluidic system consists of a polyacrylamide gel membrane (thickness ∼500 µm) and a PDMS flow cell whose channel (3 cm * 3 mm * 91 µm) contains evenly spaced square pillars (400 µm, spaced by 600 µm) to ensure a uniform pressure on the membrane. The flow was controlled by a manual valve (Hamilton, HV 4-4) connected to two syringe pumps (ProSense, NE-1000 and NE-300) by polyethylene tubing of 0.58 mm internal diameter (Smiths medical International Ltd.). The flow rate was maintained at a constant value of 20 µL.min^−1^ throughout the experiments. The polyacrylamide gel membrane was formed by mixing 1.25 mL 40% acrylamide (Bio-Rad), 3.7 mL deionized sterile water, 50 µL 10% ammonium persulfate (Sigma) and 5 µL TEMED (Bio-Rad). 450 µL of the mixture were poured in a mold consisting of a cavity aluminum slide glued to a silanized glass slide with silicon grease. A silanized glass coverslip was deposited on top and the solution was left to polymerize for about 1.5 h. After polymerization, the gel was cut in a piece of 4 * 1.5 cm and stored in a flask with sterile water.

The master mold consisted of one layer patterned by negative phototransparency masks on a silicon wafer. This layer was deposited using SU-8 (MicroChem). The PDMS flow cell was fabricated by molding silicone elastomer (Sylgard 184, Dow Corning) to this master mold. PDMS was mixed 1∶10 (v/v) ratio of catalyst and resin, poured to the master mold, degassed for 1 h and cured in an oven at 75°C for 1 h.

### Microscopy and Data Analysis

Imaging was performed with an inverted microscope (Nikon, TE2000), equipped with 100X oil objective (Nikon, Plan Fluor NA 1.3), cooled CCD camera (Photometrics, CoolSnap HQ), xenon lamp with liquid light guide (Sutter, Lambda LS), GFP filter set (Chroma, 41017), computer-controlled shutters (Sutter, Lambda 10-3 with SmartShutter) and automated stage (Märzhäuser, SCAN IM 120×100). An additional intermediate 1.5× magnification was used, resulting in images with pixel size corresponding to a length of 41 nm. The microscope control software used was MetaMorph.

Phase contrast images (300 ms exposure time with GIF filter) were taken every 75 secs; fluorescence images every 25 min (1000 ms exposure) or 15 min (500 ms exposure). Images were then analyzed with a custom Matlab program (Schnitzcells, originally provided by M. Elowitz) as detailed in Text S1. The first step consists in the detection of the cell contour for every cell in every frame. Once this segmentation step is done, a tracking step is performed to create a genealogical tree. Finally, length is measured and fluorescence is extracted. The instantaneous exponential growth rate was determined by fitting the length versus time data to an exponential function in a 12 to 24 min time window. For each experiment, 2 microcolonies were used for analysis, yielding in total 6 microcolonies.

## Supporting Information

Figure S1 **Growth of** ***E. coli*** **in minimal medium containing different concentrations of glucose as the sole carbon source (A–D).** Each curve represents the total cell length of a microcolony over time and indicates exponential growth. (E) Growth of *E. coli* in minimal medium containing no carbon source (growth on contaminants).
(TIF)

Figure S2 **Growth of** ***E. coli*** **in minimal medium containing different concentrations of lactose as the sole carbon source.** Each curve represents the total cell length of a microcolony over time and indicates exponential growth.
(TIF)

Figure S3 **Shift from a medium containing glucose and lactose to a medium containing lactose only.** (A) Example of a growth traces showing a decrease upon shift to lactose. As in the main text, the fit is shown in thick lines. (B) Example of a growth traces showing no visible growth decrease. Continuously growing cells represent ∼10% of the total lineages analyzed, which compares to the 15% obtained from glucose-only to lactose experiments.
(TIF)

Figure S4 **Example of a growth trace for a cell lineage during a shift from a glucose-only medium to a medium with no carbon source.**
(TIF)

Figure S5 **Scatter plot of duration of the lag phase (Tµ_2_−Tµ_1_) versus delays in growth decrease (ΔTµ_1_). N = 185. r^2^≈0.01 and p-value = 0.104.** No significant correlation is observed.
(TIF)

Figure S6 **Absence of lag phase is not due to cell cycle or spatial dependence.** (A) Distributions of time of shift - time of birth for cells with growth arrest (N = 105, mean = 27±12 min (SD); light grey) and continuously growing cells (N = 22, mean = 28±9.8 min (SD); dark grey), showing that the two distributions are similar. (B) Phase contrast images of microcolonies at the time of shift. Continuously growing cells are colored. The colors were chosen randomly, but sister cells were given the same color.
(TIF)

Figure S7 **Distribution of computed fluorescence induction times for cell lineages with (green) and without (red) permease present at the time of shift.**
(TIF)

Text S1 (DOC)
