# Supplementary figures and images for: Single-Cell Dynamics Reveals Sustained Growth during Diauxic Shifts
Source: PLoS One. 2013 Apr 30;8(4):e61686. doi: 10.1371/journal.pone.0061686 (PMC3640066; doi:10.1371/journal.pone.0061686)

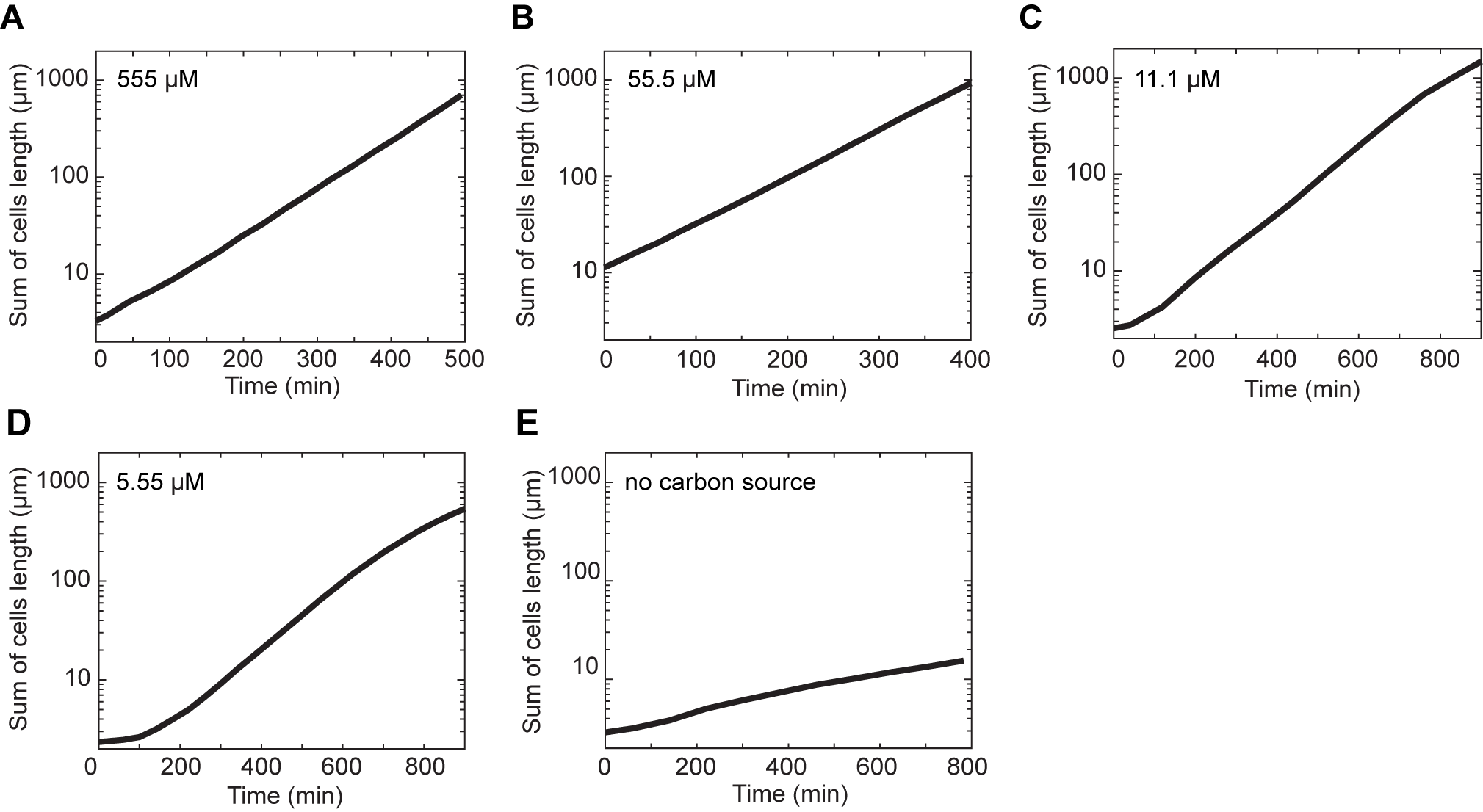

Supplement: Figure S1 — Growth of E. coli in minimal medium containing different concentrations of glucose as the sole carbon source (A–D). Each curve represents the total cell length of a microcolony over time and indicates exponential growth. (E) Growth of E. coli in minimal medium containing no carbon source (growth on contaminants). (TIF) [file pone.0061686.s001.tif]

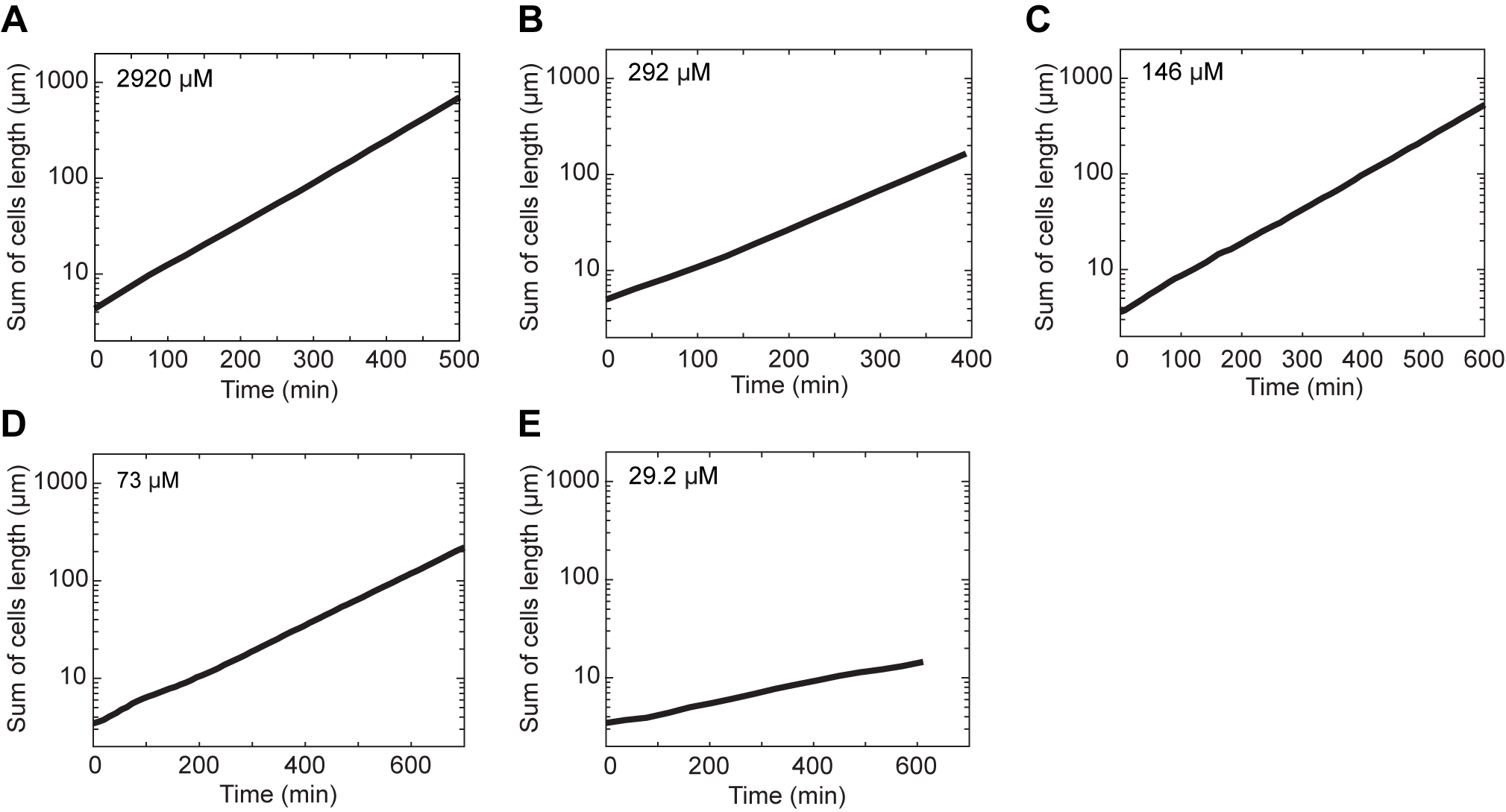

Supplement: Figure S2 — Growth of E. coli in minimal medium containing different concentrations of lactose as the sole carbon source. Each curve represents the total cell length of a microcolony over time and indicates exponential growth. (TIF) [file pone.0061686.s002.tif]

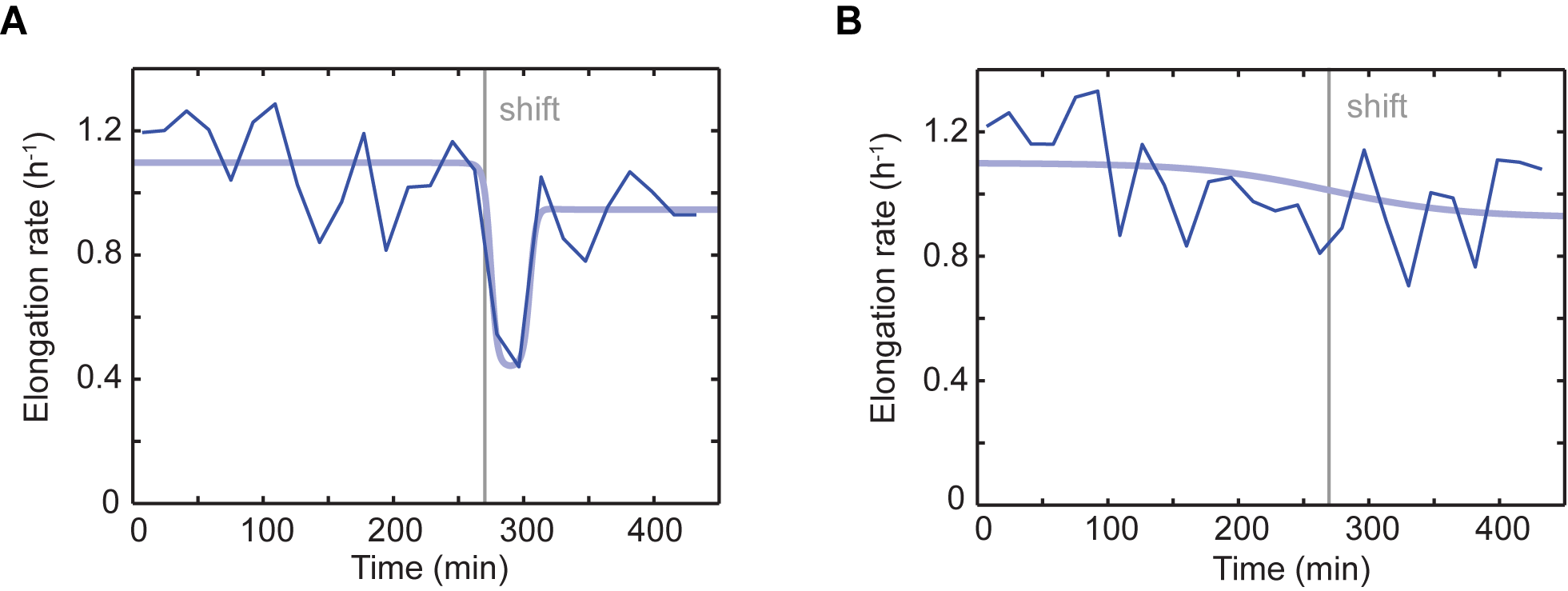

Supplement: Figure S3 — Shift from a medium containing glucose and lactose to a medium containing lactose only. (A) Example of a growth traces showing a decrease upon shift to lactose. As in the main text, the fit is shown in thick lines. (B) Example of a growth traces showing no visible growth decrease. Continuously growing cells represent ∼10% of the total lineages analyzed, which compares to the 15% obtained from glucose-only to lactose experiments. (TIF) [file pone.0061686.s003.tif]

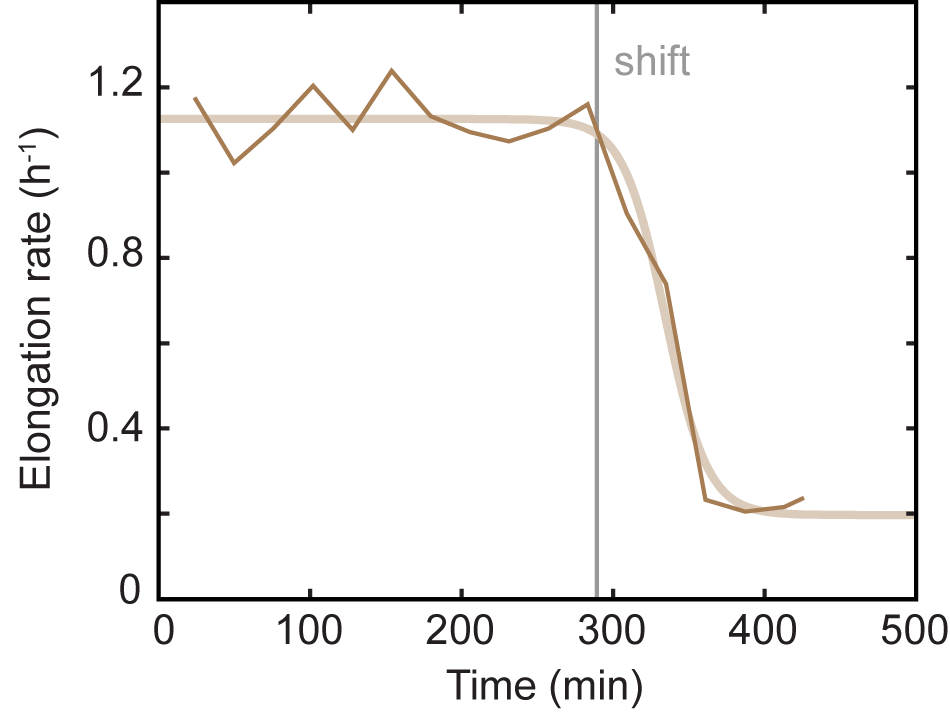

Supplement: Figure S4 — Example of a growth trace for a cell lineage during a shift from a glucose-only medium to a medium with no carbon source. (TIF) [file pone.0061686.s004.tif]

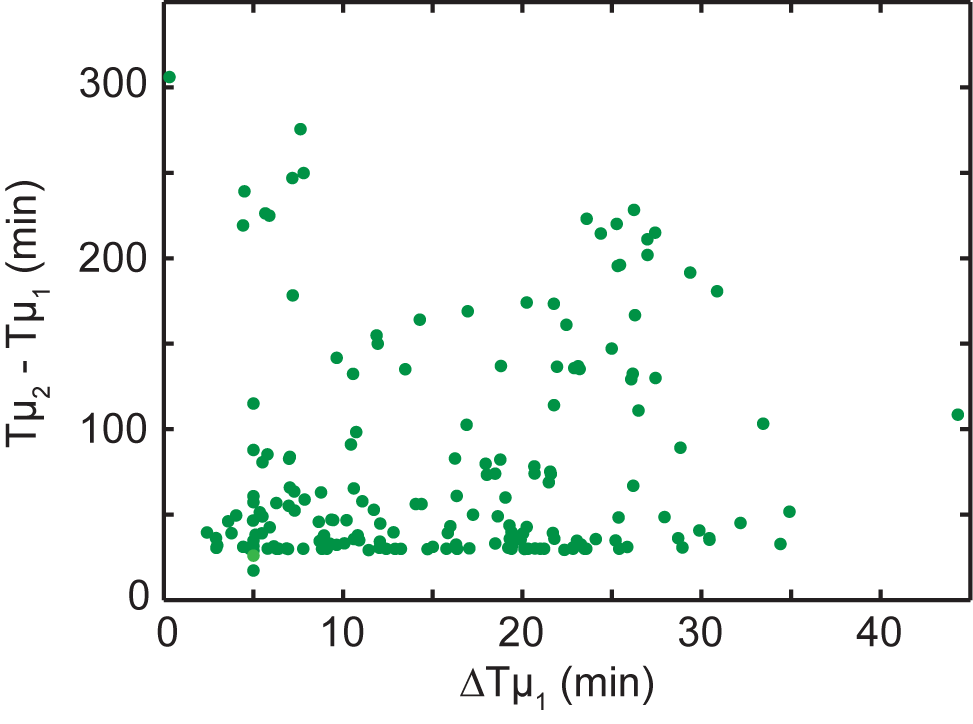

Supplement: Figure S5 — Scatter plot of duration of the lag phase (Tµ2−Tµ1) versus delays in growth decrease (ΔTµ1). N = 185. r2≈0.01 and p-value = 0.104. No significant correlation is observed. (TIF) [file pone.0061686.s005.tif]

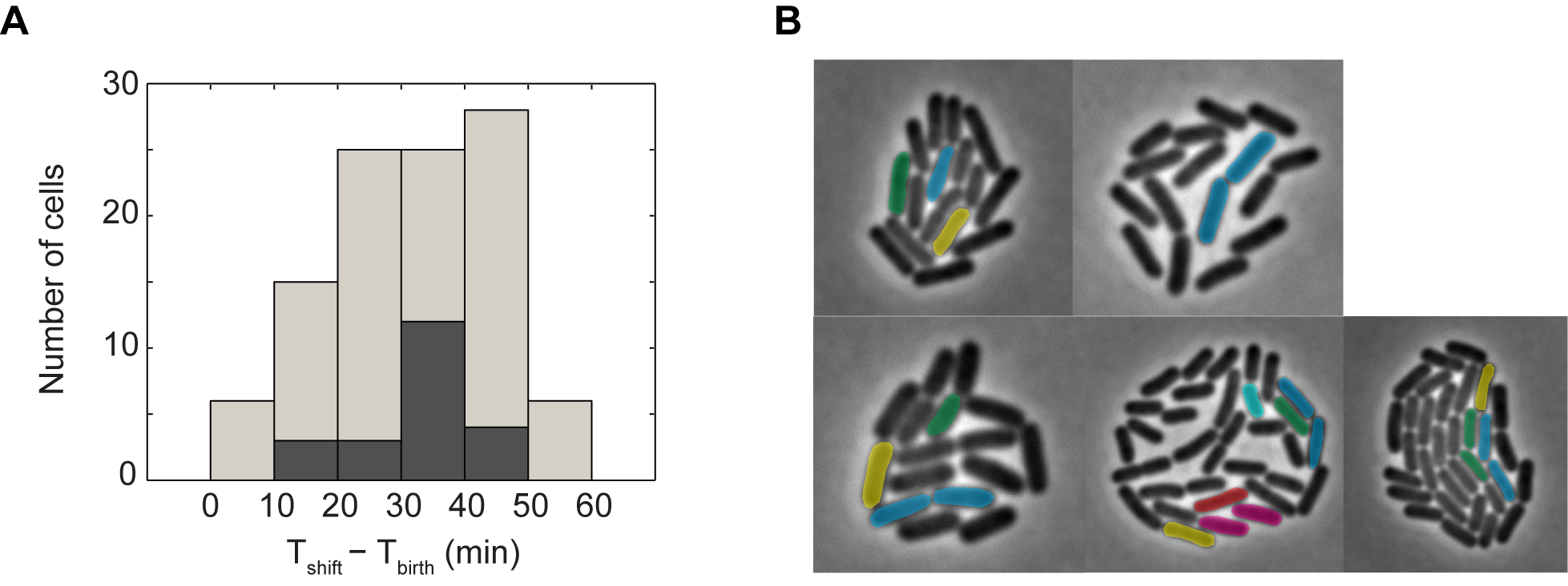

Supplement: Figure S6 — Absence of lag phase is not due to cell cycle or spatial dependence. (A) Distributions of time of shift - time of birth for cells with growth arrest (N = 105, mean = 27±12 min (SD); light grey) and continuously growing cells (N = 22, mean = 28±9.8 min (SD); dark grey), showing that the two distributions are similar. (B) Phase contrast images of microcolonies at the time of shift. Continuously growing cells are colored. The colors were chosen randomly, but sister cells were given the same color. (TIF) [file pone.0061686.s006.tif]

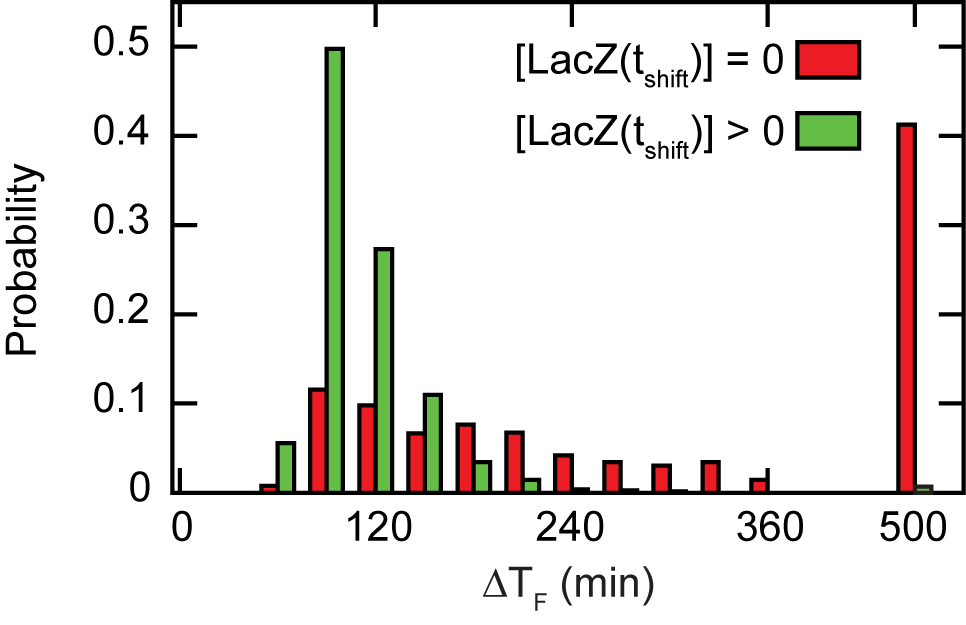

Supplement: Figure S7 — Distribution of computed fluorescence induction times for cell lineages with (green) and without (red) permease present at the time of shift. (TIF) [file pone.0061686.s007.tif]
