# Supplementary material for: Single-Cell Dynamics Reveals Sustained Growth during Diauxic Shifts
Source: PLoS One. 2013 Apr 30;8(4):e61686. doi: 10.1371/journal.pone.0061686 (PMC3640066; doi:10.1371/journal.pone.0061686)
Supplement: Text S1 — (DOC) [file pone.0061686.s008.doc]

**Single-cell dynamics reveals sustained growth during diauxic shifts**

Sarah Boulineau, Filipe Tostevin, Daniel J. Kiviet, Pieter Rein ten Wolde, Philippe Nghe and Sander J. Tans

# Supporting Information

## Data analysis

### Cell segmentation and tracking

The outline of the cells was determined by averaging the three phase contrast images (-0.2 µm, 0 µm, +0.2 µm offset from focus). From this image, edges were determined using a Laplacian of Gaussian filter. Next the background was separated from the cells, and clumps of cells were cut based on concavity and phase contrast maxima. Once each frame was segmented, lineages of cells were traced by a simple tracking algorithm that searches for nearby cells in successive frames.

### Cell length

In order to obtain an accurate length measurement, we applied the following approach. The outline of every cell is determined (during segmentation) by an edge detection that is essentially based on finding the steepest parts of intensity profiles. Next, the cell axis is determined by fitting a third degree polynomial $f(x)$ through the silhouette of the cell:

$$f(x)=ax^{3}+bx^{2}+cx+d$$

where $x$ is the main axis of the ellipsoid containing the cell. We define the length of the cell as the length of this axis from one pole ($x_{0}$) to the other ($x_{1}$). The exact pole positions along the axis are determined by taking into account the cap of the cell. The cell length ($L$) can then be obtained by numerical integration between pole $x_{0}$ and $x_{1}$ of the cell:

$$L=\int_{x_{0}}^{x_{1}} \sqrt{1+[f'(x)]^{2}dx}$$

where $f'(x)=\frac{df(x)}{dx}$. The automated length measurements within microcolonies had a precision around the pixel size of ~0.04 µm.

### Single cell growth rate

As no significant changes in cell width are observed during growth experiments, the cell size is proportional to cell length. The single cell growth rate can then be determined by a fitting the cell length over time with the function:

$$L(t)=L_{0}e^{\mu_{e}\cdot\ln(2)\cdot t}=L_{0}2^{\mu_{e}\cdot t}$$

where the elongation rate $\mu_{e}$ is effectively the same as the doubling rate. The doubling time $T_{d}$ is then:

$$T_{d}=\frac{1}{\mu_{e}}$$

The elongation rate was determined for a number of consecutive time points within a cell cycle. For independent measurements of growth rate with a high time resolution, a small window is required. However, an accurate determination of the elongation rate also requires a length increase of the cell that is significantly larger than the precision of its length measurement. Given an average birth length $L_{b}$ and the minimal required length increase $L_{i}$, the required time window $T_{w}$ is given by:

$$T_{w}=T_{d}\cdot\log_{2}\left(\frac{L_{b}+L_{i}}{L_{b}}\right)$$

We set the minimal length increase required for our time window to 0.5 µm. With an average birth length of 2 µm, this corresponds to a window of approximately a third of the cell’s doubling time.

Retrieving length data in the window around time points at the beginning or at the end of a cell cycle is problematic, as the window expands beyond the cell’s life. We applied a technique where we extend the data points for a cell’s length using the length of the mother ($L_{m}$), sister ($L_{s}$) and daughter cells ($L_{d1}$ and $L_{d2}$). At the end of a cell’s cycle additional data points are added by summation of the lengths of the two daughter cells. At the beginning of a cell’s cycle points are added by using a fraction of the length of the mother cell. Although division in *E. coli* is quite symmetric, there are still some length differences between sister cells. Therefore not exactly half of the mother cell length was taken, but a ratio based on their birth lengths. The extended length data for a cell is thus defined by:

| $L^{*}(t)=\left\{\begin{matrix} \frac{L_{0}}{L_{0}+L_{0,sister}}L_{m}(t) \\ L(t) \\ L_{d1}(t)+L_{d2}(t) \end{matrix}\right.$ | if $t<t_{0}$ |
| --- | --- |
|  | if $t_{0}\leq t\leq t_{1}$ |
|  | if $t>t_{1}$ |

### Protein concentration

Fluorescence images were corrected for camera noise and uneven illumination of the sample, using a background image ($I_{b}$) and a shading image ($I_{s}$). Given the original image $I$, the calibrated output image $I_{c}$ is given by:

$$I_{c}=\frac{I-I_{b}}{I_{s}-I_{b}}$$

The image was further enhanced by deconvolution using Matlab’s Lucy-Richardson algorithm in combination with a point-spread function for our imaging system (experimentally determined using 0.02 µm sized FluoSpheres from Invitrogen).

The total fluorescence of each cell was determined by extracting those pixels that were within the cell outline. To obtain protein concentrations from fluorescence images, one generally divides the cell’s total fluorescence by its size. The size measurement, however, is also used for calculation of the growth rate. In order to avoid artificial correlations between these two measurements, we determined the protein concentration independent of cell size. The cell mean fluorescence was calculated by averaging pixels within 0.2 μm of the cell axis, but more than 0.3 μm away from the cell poles. The protein concentration was calculated from this value by subtracting background fluorescence (determined from pixels outside the microcolony).

### Parameterized functions used to fit the experimental data

Fluorescence time traces for individual cells were fitted by the following parameterized function using the function:

$$f(t)=\frac{(b-a)\cdot\tanh\left(\left(\frac{t-t_{0}}{\tau}\right)+a+b\right)}{2}$$

where $a$ is the lower asymptote, $b$ is the upper asymptote, $\tau$ is the characteristic time of fluorescence change, $t_{0}$ is the time where fluorescence is at half its maximum ($t_{0}-t_{shift}=\Delta T_{F}$).

Similarly, elongation rates for individual cells were fitted by the following function:

$$g(t)=\left(\frac{a-b}{2}\cdot\tanh\left(\frac{t_{0}-t}{\delta}\right)+\frac{a+b-2c}{2}\right)\cdot\left(0.5+\frac{\tanh\left(\frac{abs(t-t_{0})-\delta}{\tau}\right)}{2}\right)+c$$

where $a$ is the growth rate before shift, $b$ is the growth rate after shift, $c$ is the depth of the dip, $\delta$ is the duration of the lag phase, $\tau$ is the characteristic time of growth rate change, $t_{0}$ is the time at the middle of the lag phase.

## Complete description of the stochastic model

Here we discuss in detail the stochastic model of the growth of *E. coli* and the expression of *lac* proteins during the switch of environmental conditions from glucose and lactose to lactose only. The model has been developed on the basis of a number of previously described models [1-4], but some simplifications have been made in the dynamics of protein production and sugar metabolism with many reactions coarse-grained into a single effective reaction step. Parameter values were taken from experiments where possible, and elsewhere are largely consistent with those used in these models.

### Operator dynamics and protein production

The multiple operator sites of the *lacZYA* operon and the multiple binding sites of the LacI repressor for both DNA and inducer mean that the chromosome-repressor-inducer complex can exist in many distinct binding states [5]. While it has been proposed that individual partial dissociation events can lead to observable changes in *lac* expression [6], it remains unclear which are the relevant binding configurations in vivo and how expression and binding propensities vary between different binding states. We therefore consider a greatly simplified phenomenological model of a single *lac* operator which can exist in only two states: the operator can be either BOUND by repressor, in which case there is a slow rate of leaky protein production $\kappa_{B}$, or FREE of repressor, in which case protein production occurs at a rapid rate $\kappa_{F}([glu])$. The regulatory effect of the cAMP pathway, which increases *lac* expression in environments lacking glucose, is represented through the dependence of the production rate from the FREE operator on the environmental glucose concentration, according to the relationship:

$\kappa_{F}([glu])=\frac{\kappa_{F,\max}}{1+[glu]/K_{CAP}}$ (S1)

The effective parameter , the effective repression strength of glucose, incorporates a number of processes including cAMP expression, cAMP-CRP binding and binding of CAP to the DNA, and should not be interpreted as describing any specific molecular interaction. We do not include any effect of cAMP on the transcription rate in the BOUND state as we assume that the timescale of these bursts is determined primarily by partial dissociations of repressor in the actual system (see below), which we take to be independent of CAP binding. We additionally assume that the rate of protein production events is dependent on the growth rate of the cell, since under starvation conditions cells may be limited in their ability to synthesis proteins. Specifically, we assume that the rate of production events in a cell with growth rate $\mu$ is modulated by a factor $2\mu/(\mu_{\max}+\mu)$, where $\mu_{\max}$ is the maximal achievable rate of growth on glucose.

For simplicity we assume that all proteins in the *lac* operon are coexpressed, and represent the concentration of both LacZ and LacY proteins by $[Z]$. Protein concentrations are calculated from the copy number $N_{Z}$ and cell length $l$ assuming a cylindrical cell with a constant radius of r = 0.25 μm.

Statistics of *lac* protein production have been measured in [6, 7]. They described two types of production bursts. During small bursts the number of proteins produced, $b$, is exponentially distributed,

$p(b)=\frac{e^{-b/\bar{b}}}{}e^{1/\bar{b}}-1$ (S2)

with $\bar{b}$~5. This was interpreted as the distribution of proteins produced from a single mRNA transcript. Larger bursts were also found, although their distribution was not characterized. We interpret these large bursts as resulting from many mRNA transcripts being produced in quick succession, such that the products of the individual transcripts cannot be resolved. Here, we assume that an individual gene transcription event, in either the BOUND or FREE operator state, gives rise to a number of proteins according to the distribution (S2). The production characteristics in these two states differ only in the average rate of transcription events.

We assume that LacI repressor proteins are produced at a rate $\kappa_{R}$. The concentration of repressors decreases only due to dilution, with no degradation. Repressors can be active or inactive; binding of inducer causes repressor deactivation. Since repressor-inducer binding and dissociation is fast ($\leq$ 1s) we do not model (de)activation reactions explicitly but instead assume that this is a reversible reaction at equilibrium. The fraction of active repressors is then given by:

$\frac{[R]^{*}}{[R]_{T}}=\frac{1}{1+([L]/K_{R})^{h}}$ (S3)

where $[R]_{T}=N_{R}/l$ is the total concentration of repressor proteins, $N_{R}$ is the number of repressor proteins in the cell, $[L]$ is the intracellular lactose concentration and $K_{R}$ is the binding constant for lactose (or products of lactose metabolism) to the repressor. The Hill coefficient $h$ allows for cooperativity in the deactivation of the repressor tetramer. The rate of association of repressor to the FREE operator depends on the concentration of active LacI, $k_{F\to B}[R]^{*}$. We assume that dissociation of LacI from the operator is also enhanced by the presence of lactose. We assume that at any time a fraction $\phi=(1+K_{R}^{'}/[L])^{-1}$ of DNA-bound repressors is associated with inducer. The net dissociation rate of repressors from the operator is then taken to be $k_{B\to F}(1+k^{'}\phi)$, where $k^{'}$ is the enhancement factor of dissociation for inducer-bound repressors. We emphasize that since we use a simplified two-state operator model, this enhancement of dissociation represents an effective action of lactose on repressor binding, which in reality could be due to a number of processes on a molecular level.

#### Parameter values

The maximal rate of production bursts for the FREE operator is taken to be $\kappa_{F,\max}$= 15 min^-1^, comparable to the experimentally-determined rate of transcription initiation [8]. This leads to copy numbers of a few thousand molecules in fully induced cells, which is also consistent with data of [6]. For the leaky production rate when the operator is BOUND we take the burst rate measured in [6, 7] for fully repressed cells, $\kappa_{B}$*~*0.5 per cell cycle ~0.005 min^-1^. The strength of cAMP-mediated transcriptional repression by glucose, $K_{CAP}$ = 30 μM, is chosen to fit the results to the experimental delay distributions.

The typical concentration of repressors is 10-50 nM, corresponding to ~1-20 per cell [9, 10]. We take a production rate $\kappa_{R}=5\cdot\mu$, which gives a typical concentration of $[R]_{T}$ ≈ 20 nM.

The choice of binding constant for inducer-inhibitor binding is complicated by the fact that it is allolactose, rather than lactose itself, which binds to the repressor, but our model does not include allolactose explicitly. However, in practice upon the addition of lactose cells rapidly reach a stable high lactose concentration $\geq$1 mM, and the precise value of the parameter $K_{R}$ will have little effect on the dynamics provided that it is much smaller than this value. We simply take $K_{R}$ = 10 μM, comparable to measured values for allolactose and other strong inducers [11]. The effective Hill coefficient for deactivation of inhibitor has been estimated as $h$ *≈* 2 [12]. For the binding constant for inducer and DNA-bound repressor we take $K_{R}^{'}$ = 200 μM.

For the binding rate of repressor to the operator we use $k_{F\to B}$ = 60 μM^-1^min^-1^, which gives a binding rate comparable to the association time measured in [9]. A dissociation rate of LacI from the operator of $k_{B\to F}$ = 0.0005 min^-1^ gives rise to an ~1100-fold difference between the mean expression levels in the repressed and fully-induced states, which is again consistent with the data of [6]. Experiments suggest that the enhancement of this dissociation rate by inducer for binding to a single operator fragment can be as large as a factor of 1000 [13]. Here we choose $k^{'}$ = 100, since in the presence of DNA looping with multiple operator binding sites not all dissociation events will lead to full release of the repressor.

### Intracellular lactose dynamics

We do not consider individual lactose molecules since these will be present in extremely high copy numbers within the cell. The rate of import of extracellular lactose into the cell depends on the concentration of permeases, which equals the concentration of LacZ, their activity, and the external lactose concentration:

$\upsilon_{import}=\rho([glu])\frac{[Z][L]_{ex}}{K_{import}+[L]_{ex}}$ (S4)

Following previous models [1, 3], inducer exclusion is implemented by making the import rate for an individual permease a decreasing function of the environmental glucose concentration, $\rho([glu])=\rho_{\max}/(1+[glu]/K_{excl})$. Intracellular lactose is diluted during growth. In addition, it is metabolized by LacZ. Since typically $[L]>>[Z]$, the flux of this reaction is simply taken to be proportional to $[Z]$,

$\upsilon_{consumption}=\Delta\frac{[Z][L]}{K_{L}+[L]}$ (S5)

Finally, therefore, the overall lactose concentration within a cell follows:

$\frac{d[L]}{dt}=\upsilon_{import}-\upsilon_{consumption}-\mu[L]$ (S6)

where $\mu$ is the cell growth rate.

#### Parameter values

The maximal import rate of lactose is around $\rho_{\max}$ = 2000 min^-1^ [14, 15]. The critical glucose concentration for inducer exclusion is determined, together with $K_{CAP}$*,* by fitting the delay time distributions and is taken to be $K_{excl}$ = 30 μM. The lactose import saturation concentration is $K_{import}$ = 400 μM [14, 15]. The maximal rate of metabolism of lactose by a single β-galactosidase enzyme is set as $\Delta$ = 3600 min^-1^ [16]. The saturation coefficient for lactose conversion by β-galactosidase is $K_{L}$ = 1.4 mM [16].

### Growth rate

There are three sources of growth in the experiments: glucose, lactose and contaminants, and these can all be present simultaneously. In order to model the combined metabolism of all three carbon sources at once we assume that these three sources contribute to the same effective metabolic flux,

$\Phi=[glu]+\delta\frac{[Z][L]}{K_{L}+L}+\Phi_{res}$ (S7)

Here the first term is the concentration of glucose in the environment. The metabolism of glucose is not simulated in detail, and is simply assumed to be independent of the internal state of the cell. The second term in (S7) is the flux of lactose metabolism by LacZ. The parameter $\delta$ weighs the relative contributions of glucose and lactose metabolism. Finally, $\Phi_{res}$ is a constant residual flux due to contaminants.

This growth rate is then taken to be a Monod-function of the flux,

$\mu=\mu_{\max}\frac{\Phi}{\Phi+K_{\Phi}}$ (S8)

The parameter $K_{\Phi}$ reflects the fact that growth is limited by other processes independent of metabolism, and therefore cannot be increased arbitrarily.

#### Parameter values

The parameters $\mu_{mac}$ = 1.02 h^-1^, $\Phi_{res}$ = 0.54 μM and $K_{\Phi}$ = 5.1 μM can be found by fitting the growth rate as a function of glucose level in the absence of lactose. We choose $\delta$ = 50 to fit the experimental growth dynamics.

A consequence of assuming this form for the growth rate is that the maximal growth rate on lactose can approach the maximal growth rate on glucose if the lactose concentration $[L]>K_{L}$ and $\delta[Z]>K_{\Phi}$. With the parameters listed above the maximal expression level is $[Z]$ ≈ 25 μM, and hence the maximal growth rates on the two substrates are similar.

### Cell division

The change in cell length over time follows:

$\frac{dl}{dt}=\mu l$ (S9)

For a constant growth rate $\mu$, this corresponds to exponential growth. We assume that all cells divide when they reach a critical length $l_{div}$, which is independent of the growth rate or protein levels. Upon division a new cell is created with half of the length of the parent cell, which also has its length halved. The repressor and LacZ molecules in the parent cell are binomially-partitioned between the parent and daughter cells (i.e. each protein is moved into the new daughter cell with probability 0.5 or otherwise remains in the parent cell). Assuming that the number of lactose molecules is large and noise in partitioning can be neglected, the lactose concentration in the daughter cell is set to be the same as that of the parent cell. The daughter cell also inherits a copy of the operator which is in the same state (BOUND or FREE) as the parent at the time of division.

#### Parameter values

$l_{div}$ = 3 μm.

### Simulation protocol

The model is propagated with a constant time step $\delta t$. At each time step the active repressor and LacZ concentrations and the growth rate are recalculated according the current cell length and lactose concentration. $[L]$and $l$ are updated according to Eqs. (S6) and (S9) with a first-order difference scheme (i.e. $l(t+\delta t)=l(t)+\delta t\cdot l^{'}(t)$). Switching of the operator state is treated probabilistically: the operator can switch with probability $\delta t\cdot k$, where $k$ is the relevant switching rate. Similarly, a protein production burst takes place with probability $\delta t\cdot\kappa$, where $\kappa$ is the appropriate production rate for the current operator state. Errors due to this discretization will be negligible provided $\delta t$ is chosen to be small enough. Here we take $\delta t$ = 0.001 min, which ensures that the probability for each stochastic reaction to occur during each time step in much less than 1.

First, an initialization simulation is performed. A population of 2000 cells is simulated in the presence of glucose only, $[glu]$ = 555 μM and $[L]_{ex}$ = 0, for a period of 10000 minutes. During this simulation only one of the daughter cells produced in each cell division event is retained, such that the population size is constant and each cell present at the end of the simulation is an independent sample of the steady-state distribution of cellular states.

Next, a sub-population of 100 cells is selected randomly from those present at the end of initialisation simulation to form the initial population in the simulation of the experimental conditions. During the subsequent phases of simulation all daughter cells are retained and propagated to the end of the simulation period in order to preserve correlations between related lineages. This population of cells is then propagated as described above in an environment mimicking the experimental conditions of glucose, $[glu]$ = 555 μM, and lactose, $[L]_{ex}$ = 3mM, until the shift time $t_{shift}$ = 210 min. After the shift time the concentration of glucose is decreased according to $[glu](t)=\exp[-(t-t_{shift})/\tau]\cdot555\mu M$, while the concentration of lactose remains unchanged. The timescale $\tau$ = 5 min is chosen to match the experimentally-determined timescale for diffusion out of the flow chamber. The simulation continues until a time $t$ = 610 min.

For each cell, time series of the growth rate and expression level data are recorded, and subsequently used to recreate the trajectory of each cell lineage. The mean concentration in fully-induced cells in the presence of lactose is $[Z]$ ≈ 25 μM. Lineages which reach a concentration level of at least $[Z]$ = 17 μM during the simulation are identified as becoming induced. For each such lineage the threshold LacZ concentration for calculating ΔT_F_ is set at half the maximal concentration of the lineage. Lineages for which the growth rate never decreases below $\mu$ = 0.82 h^-1^ are designated as having no lag phase. For the remaining lineages, the time of growth decrease ΔTµ_1_ is found as the time at which the growth rate trajectory crosses a threshold half-way between the maximal growth rate on glucose and the lowest growth rate after the shift of medium. The time of growth recovery ΔTμ_2_ is similarly found as the time at which the growth rate crosses a threshold midway between the lowest growth rate after the shift and the highest subsequent growth rate.

# Supporting Notes

## Note 1: Cell size during lag phase

For the first generation of cells in the lag phase (born before the lag phase), the division time was not strongly affected by the significant reduction in growth rate. Consequently, the cell size at the first division in the lag phase decreased (from 3.2 µm to 2.8 µm on average). In contrast the cells being born and dividing during the lag phase have an increased division time, matching the low growth rate. These observations suggest that the doubling time is set early on in the cell cycle. A possible mechanism could be that the division time is determined primarily by the start of chromosome replication, which occurs at the beginning of the cell cycle.

# Supporting References

[1] Wong P, Gladney S, Keasling JD (1997) Mathematical model of the *lac* operon: inducer exclusion, catabolite repression, and diauxic growth on glucose and lactose. Biotechnol Prog 13:132-143.

[2] Yildrim N, Mackey MC (2003) Feedback regulation in the lactose operon: a mathematical modeling study and comparison with experimental data. Biophys J 84:2841-2851.

[3] Santillán M, Mackey MC (2004) Influence of catabolite repression and inducer exclusion on the bistable behavior of the *lac* operon. Biophys J 86:1282-1292.

[4] Kalisky T, Dekel E, Alon U (2007) Cost-beneﬁt theory and optimal design of gene regulation functions. Phys Biol 4:229-245.

[5] Oehler S, Eismann ER, Krämer H, Müller-Hill B (1990) The three operators of the *lac* operon cooperate in repression. EMBO J 9:973-979.

[6] Choi PJ, Cai L, Frieda K, Xie XS (2008) A stochastic single-molecule event triggers phenotype switching of a bacterial cell. Science 322:442-446.

[7] Cai L, Friedman N, Xie XS (2006) Stochastic protein expression in individual cells at the single molecule level. Nature 440:358-362.

[8] Kennell D, Reizman H (1977) Transcription and translation initiation frequencies of the *Escherichia coli lac* operon. J Mol Biol 114:1-121.

[9] Elf J, Li GW, Xie XS (2007) Probing transcription factor dynamics at the single-molecule level in a living cell. Science 316:1191-1194.

[10] Gilbert W, Müller-Hill B (1966) Isolation of the *lac* repressor. Proc Natl Acad Sci U S A 56:1891-1898.

[11] Barkley ND, Riggs AD, Jobe A, Bourgeois S (1975) Interaction of eﬀecting ligands with *lac* repressor and repressor-operator complex. Biochemistry 14:1700-1712.

[12] Yagil G, Yagil E (1971) On the relation between eﬀector concentration and the rate of induced enzyme synthesis. Biophys J 11:11-27.

[13] Dunaway M, Olson JS, Rosenberg JM, Kallai OB, Dickerson RE, et al. (1980) Kinetic studies of inducer binding to *lac* repressor-operator complex. J Biol Chem 255:10115-10119.

[14] Wright JK, Riede I, Overath P (1981) Lactose carrier protein of *Escherichia coli*: interaction with galactosides and protons. Biochemistry 20:6404-6415.

[15] Lolkema JS, Carrasco N, Kaback HR (1991) Kinetic analysis of lactose exchange in proteoliposomes reconstituted with puriﬁed *lac* permease. Biochemistry 30:1284-1290.

[16] Martinez-Bilbao M, Holdsworth RE, Edwards LA, Huber RE (1991) A highly reactive β-galactosidase (*Escherichia coli*) resulting from a substitution of an aspartic acid for Gly=794*. J Biol Chem 266:4979-4986.
